# Supplementary material for: T follicular helper and T follicular regulatory cells have different TCR specificity
Source: Nat Commun. 2017 Apr 21;8:15067. doi: 10.1038/ncomms15067 (PMC5413949; doi:10.1038/ncomms15067)
Supplement: Supplementary Information — Supplementary Figures and Supplementary Tables [file ncomms15067-s1.pdf]

Supplementary Figures

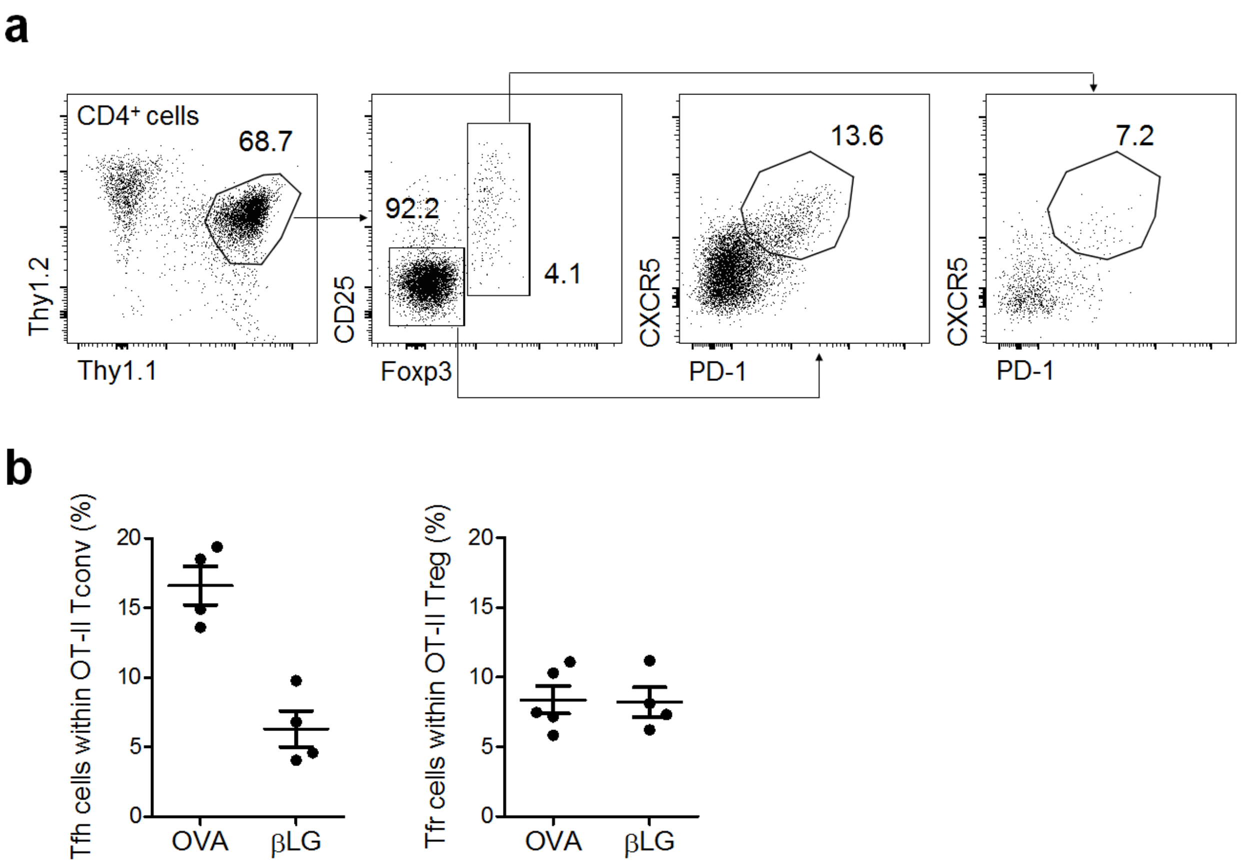

Supplementary Figure 1. *OT-II.Rag^{+}Treg cells differentiate into Tfr cells independently of the immunization and keep Vα2 and Vβ5 expression.* (**a**) Gating strategy to determine the percentage of OT-II Treg and Tconv cells that differentiate into Tfr and Tfh cells, respectively. (**b**) While the percentage of OT-II Tconv that differentiate into Tfh is higher in mice immunized with OVA than βLG (left), the same percentage of OT-II Treg originates Tfr cells in both immunizations (right). Mean ± SEM presented for n=5.

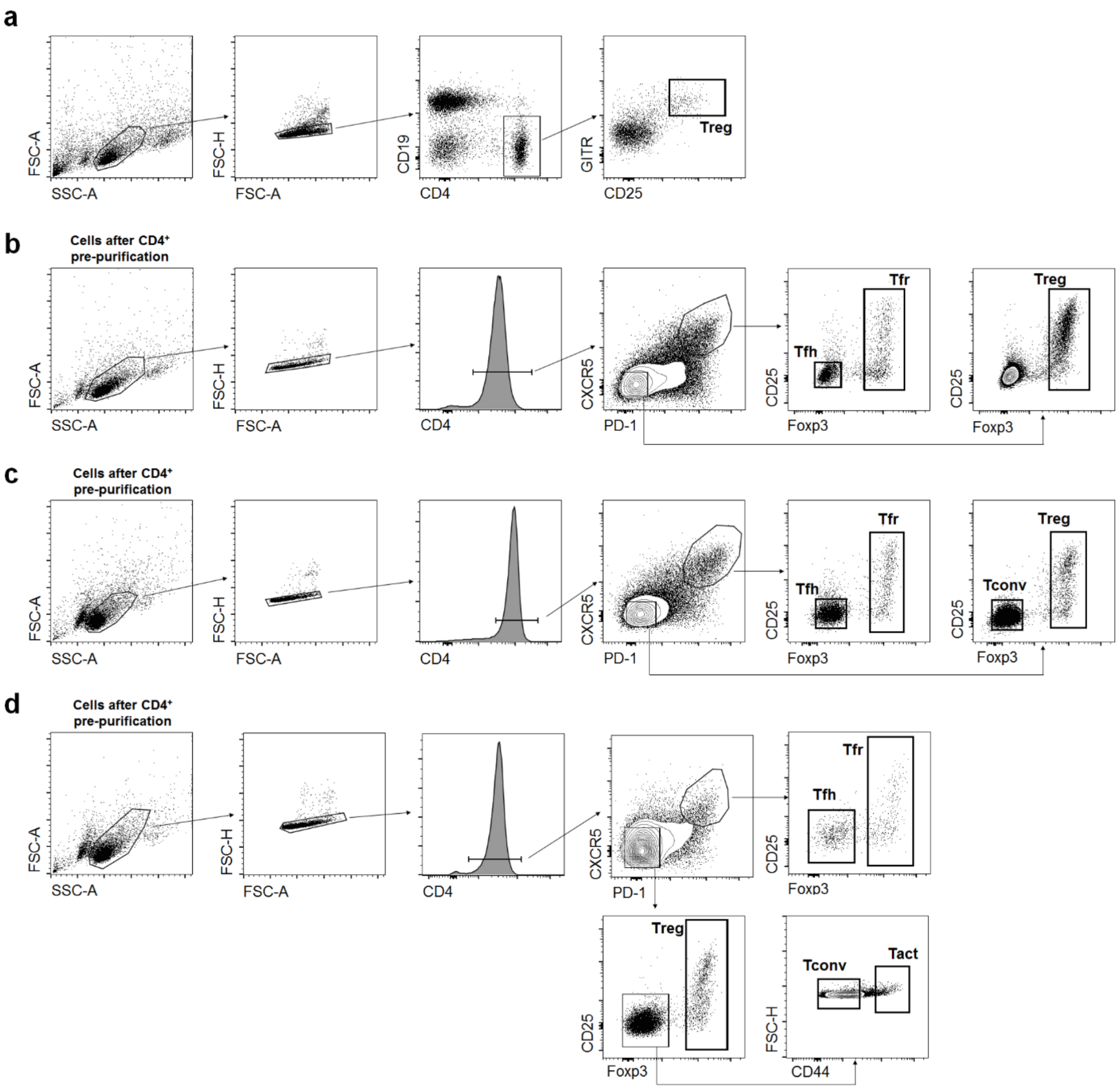

Supplementary Figure 2. *Gating strategies used for cell sorting.* (**a**) Gating strategy to sort Treg (CD4^{+}CD25^{+}GITR^{+}) cells from OT-II.*Rag*$^{+}$ and C57BL/6 WT mice for *in vitro* cultures presented on Fig. 2g,h. (**b**) Gating strategy to sort Tfh (CD4^{+}CXCR5^{+}PD-1^{+}Foxp3^{-}), Tfr (CD4^{+}CXCR5^{+}PD-1^{+}Foxp3^{+}), and Treg (CD4^{+}CXCR5^{-}PD-1^{-}Foxp3^{+}) cells from *Foxp3*^{hCD2} mice used on the *in vitro* proliferation assay presented on Fig. 4f-h. (**c**) Gating strategy to sort Tfh (CD4^{+}CXCR5^{+}PD-1^{+}Foxp3^{-}), Tfr (CD4^{+}CXCR5^{+}PD-1^{+}Foxp3^{+}), Treg (CD4^{+}CXCR5^{-}PD-1^{-}Foxp3^{+}), and Tconv (CD4^{+}CXCR5^{-}PD-1^{-}Foxp3^{-}) cells from *Foxp3*^{gfp} mice for *TRBV* CDR3 spectratyping/Immunoscope analysis (Fig. 5). (**d)** Gating strategy to sort Tfh (CD4^{+}CXCR5^{+}PD-1^{+}Foxp3^{-}), Tfr (CD4^{+}CXCR5^{+}PD-1^{+}Foxp3^{+}), Treg (CD4^{+}CXCR5^{-}PD-1^{-}Foxp3^{+}), Tact (CD4^{+}CXCR5^{-}PD-1^{-}Foxp3^{-}CD44^{+}), and Tconv (CD4^{+}CXCR5^{-}PD-1^{-}Foxp3^{-}CD44^{-}) cells from 1D2β mice for *TRA* sequencing analysis (Fig. 6c-g) The same strategy was used to sort Tfh cells for the *in vitro* proliferation assay presented on Fig. 6b.

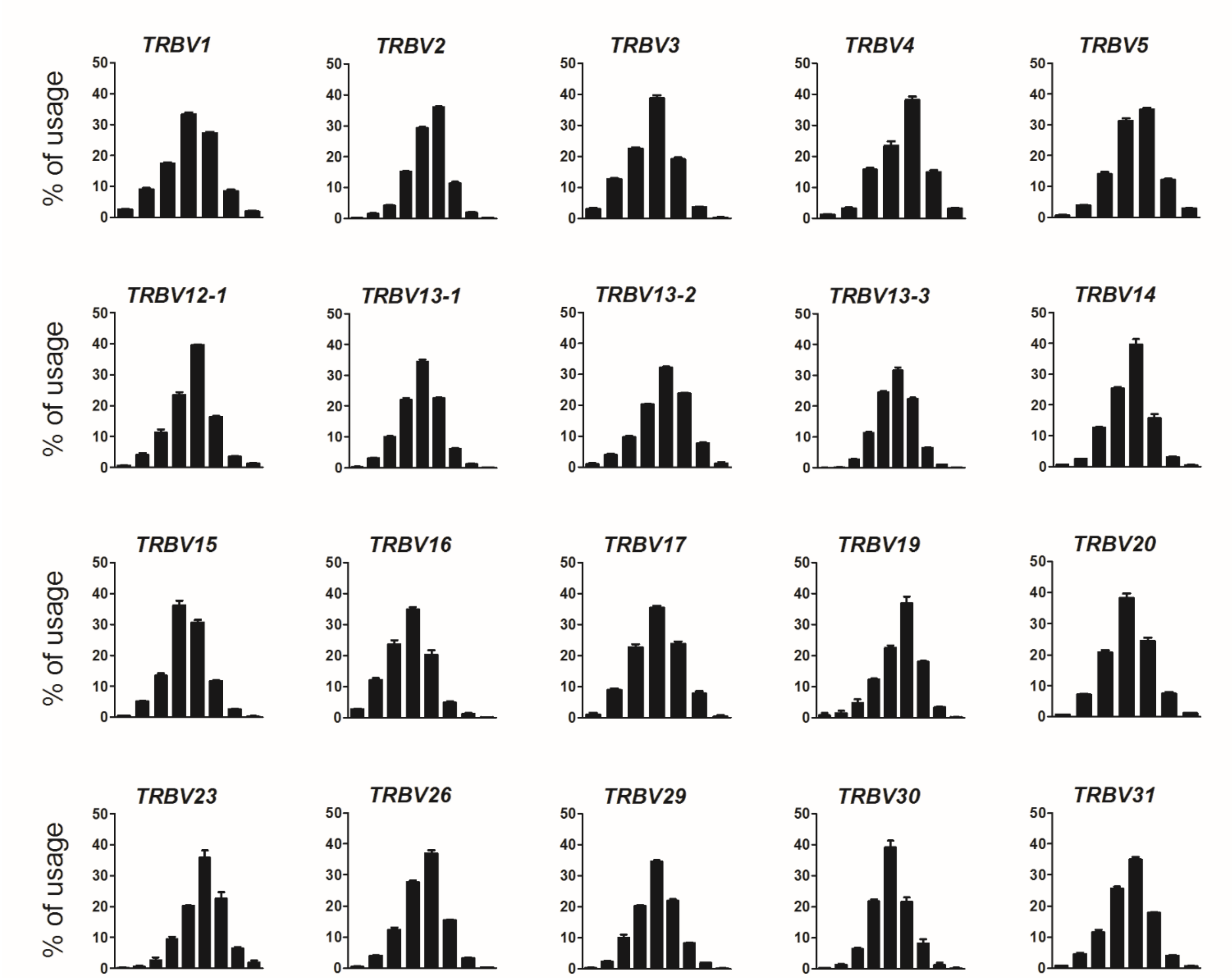

Supplementary Figure 3. *CDR3 spectratypes obtained from naïve CD4⁺ T cells.* CDR3-length usage distribution of 20 *TRBV* segments from CD4⁺ T cells from naïve mice that present a Gaussian-like distribution. Bar graphs present Mean ± SEM of four samples.

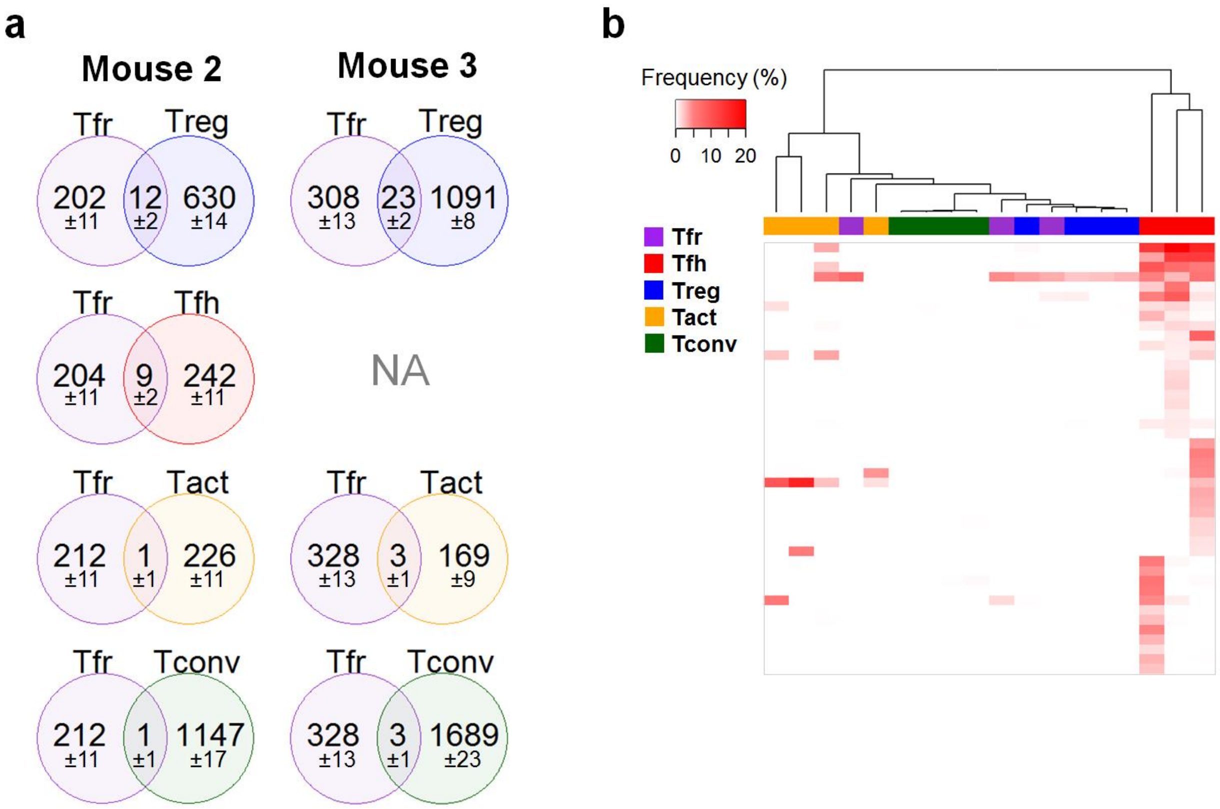

Supplementary Figure 4. *Common clonotypes for Tfr cells and 20 most predominant clonotypes of Tfh samples*. (**a**) Venn diagrams of the shared clonotypes between Tfr and other populations for mouse 2 and 3. Numbers presented are the Mean ± SD of clonotypes identified after 100 iterations of the sampling process. As it was observed on Fig. 6, Tfr cells share more clonotypes with Treg cells. Sequencing results for Tfh sample of mouse 3 are not available (NA). (**b**) Heatmap and hierarchical clustering of the 20 most predominant clonotypes for each Tfh sample. The predominant clonotypes are mainly shared between Tfh samples and Tact samples.

Supplementary Tables

Supplementary Table 1. Pairwise Multiple Comparison Analysis with Holm-Bonferroni Correction between Samples TRBV Perturbation Scores.

	Tconv1	Tconv2	Tconv3	Tconv4	Tfh1	Tfh2	Tfh3	Tfr1	Tfr2	Tfr3	Treg1	Treg2
**Tconv2**	1	-	-	-	-	-	-	-	-	-	-	-
**Tconv3**	1	1	-	-	-	-	-	-	-	-	-	-
**Tconv4**	1	1	1	-	-	-	-	-	-	-	-	-
**Tfh1**	0,014 *	0,015 *	0,033 *	0,016 *	-	-	-	-	-	-	-	-
**Tfh2**	0,000 ***	0,000 ***	0,000 ***	0,000 ***	0,673	-	-	-	-	-	-	-
**Tfh3**	0,007 **	0,009 **	0,012 *	0,009 **	1	0,473	-	-	-	-	-	-
**Tfr1**	1	1	1	1	0,017 *	0,000 ***	0,007 **	-	-	-	-	-
**Tfr2**	1	1	1	1	0,162	0,000 ***	0,101	0,495	-	-	-	-
**Tfr3**	1	1	1	1	0,002 **	0,000 ***	0,002 **	1	1	-	-	-
**Treg1**	1	1	1	1	0,046 *	0,000 ***	0,004 **	1	1	1	-	-
**Treg2**	1	1	1	1	0,037 *	0,000 ***	0,018 *	1	1	1	1	-
**Treg3**	1	1	1	1	0,007 **	0,000 ***	0,007 **	1	1	1	1	1

p<0.05 *, p<0.01 **, p<0.001 ***

Supplementary Table 2. List of Primers Used on This Study.

Sequence	Target gene/region	Orientation	Application
TCACTGATACGGAGCTGAGGC	*TRBV1*	Forward	Spectratyping
GCCTCAAGTCGCTTCCAACCTC	*TRBV2*	Forward	Spectratyping
CACTCTGAAAATCCAACCCAC	*TRBV3*	Forward	Spectratyping
ATCAAGTCTGTAGAGCCGGAGGA	*TRBV4*	Forward	Spectratyping
CTGAATGCCCAGACAGCTCCAAGC	*TRBV5*	Forward	Spectratyping
AAGGTGGAGAGAGACAAAGGATTC	*TRBV12-1*	Forward	Spectratyping
CATTATGATAAAATGGAGAGAGAT	*TRBV12-2*	Forward	Spectratyping
TGCTGGCAACCTTCGAATAGGA	*TRBV13-1*	Forward	Spectratyping
CATTATTCATATGGTGCTGGC	*TRBV13-2*	Forward	Spectratyping
CATTACTCATATGTCGCTGAC	*TRBV13-3*	Forward	Spectratyping
AGGCCTAAAGGAACTAACTCCAC	*TRBV14*	Forward	Spectratyping
GATGGTGGGGCTTTCAAGGATC	*TRBV15*	Forward	Spectratyping
GCACTCAACTCTGAAGATCCAGAGC	*TRBV16*	Forward	Spectratyping
TCTCTCTACATTGGCTCTGCAGGC	*TRBV17*	Forward	Spectratyping
CTCTCACTGTGACATCTGCCC	*TRBV19*	Forward	Spectratyping
CCCATCAGTCATCCCAACTTATCC	*TRBV20*	Forward	Spectratyping
CTGCTAAGAAACCATGTACCA	*TRBV21*	Forward	Spectratyping
TCTGCAGCCTGGGAATCAGAA	*TRBV23*	Forward	Spectratyping
AGTGTTCCTCGAACTCACAG	*TRBV24*	Forward	Spectratyping
CCTTGCAGCCTAGAAATTCAGT	*TRBV26*	Forward	Spectratyping
TACAGGGTCTCACGGAAGAAGC	*TRBV29*	Forward	Spectratyping
CAGCCGGCCAAACCTAACATTCTC	*TRBV30*	Forward	Spectratyping
ACGACCAATTCATCCTAAGCAC	*TRBV31*	Forward	Spectratyping
GCCCATGGAACTGCACTTGGC	*TRBC*(1)	Reverse	Spectratyping
FAM-CTTGGGTGGAGTCACATTTCTC	*TRBC*(2) (spectratyping run-off)	Reverse	Spectratyping
CAACGCAGAGTGGCCATTAC	Mint-2 universal adapter	Forward	Sequencing
GCAGGTGAAGCTTGTCTGGT	*TRAC*	Reverse	Sequencing
